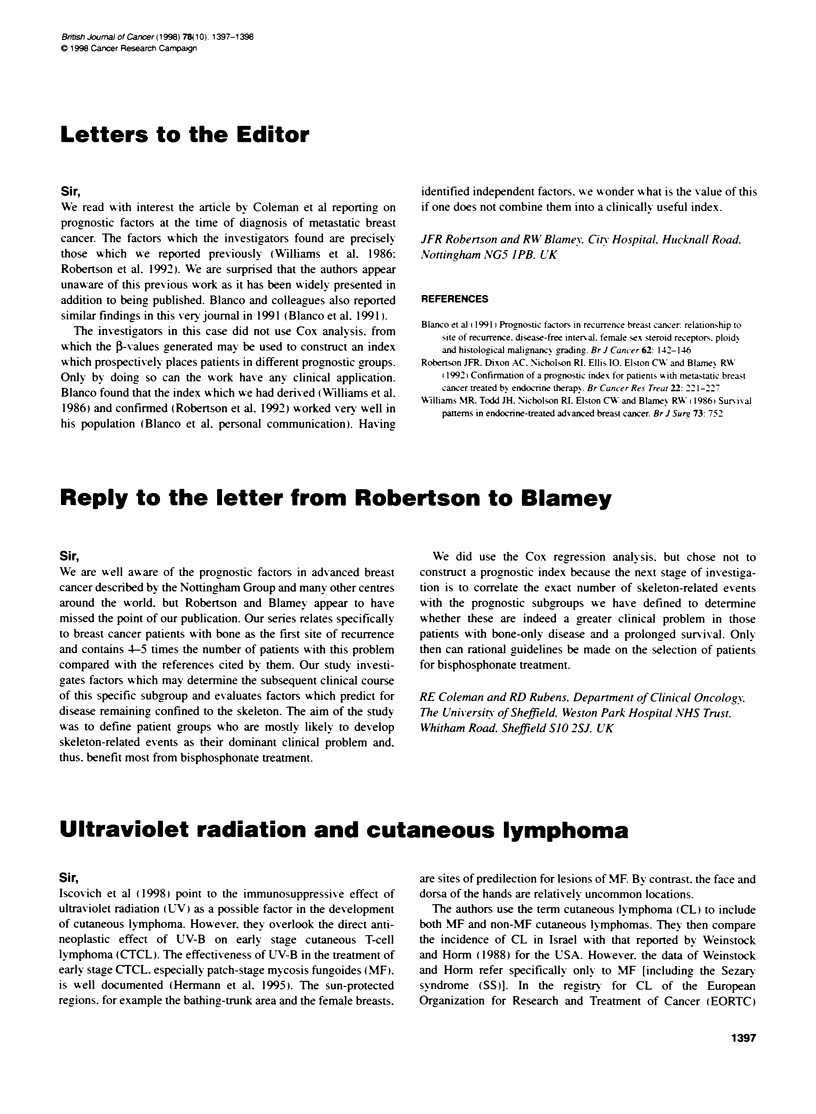# Prognostic factors at the time of diagnosis of metastatic breast cancer.

**DOI:** 10.1038/bjc.1998.692

**Published:** 1998-11

**Authors:** J. F. Robertson, R. W. Blamey


British Journal of Cancer (1998) 78(10), 1397–1398

© 1998 Cancer Research Campaign

### Letters to the Editor

**Sir,**

We read with interest the article by Coleman et al reporting on prognostic factors at the time of diagnosis of metastatic breast cancer. The factors which the investigators found are precisely those which we reported previously (Williams et al. 1986: Robertson et al. 1992). We are surprised that the authors appear unaware of this previous work as it has been widely presented in addition to being published. Blanco and colleagues also reported similar findings in this very journal in 1991 (Blanco et al. 1991).

The investigators in this case did not use Cox analysis, from which the β-values generated may be used to construct an index which prospectively places patients in different prognostic groups. Only by doing so can the work have any clinical application. Blanco found that the index which we had derived (Williams et al. 1986) and confirmed (Robertson et al. 1992) worked very well in his population (Blanco et al. personal communication). Having identified independent factors, we wonder what is the value of this if one does not combine them into a clinically useful index.

*JFR Robertson and RW Blamey, City Hospital, Hucknall Road, Nottingham NG5 1PB, UK*

### REFERENCES

Blanco et al (1991) Prognostic factors in recurrence breast cancer: relationship to site of recurrence, disease-free interval, female sex steroid receptors, ploidy and histological malignancy grading. *Br J Cancer* **62**: 142–146

Robertson JFR, Dixon AC, Nicholson RI, Ellis IO, Elston CW and Blamey RW (1992) Confirmation of a prognostic index for patients with metastatic breast cancer treated by endocrine therapy. *Br Cancer Res Treat* **22**: 221–227

Williams MR, Todd JH, Nicholson RI, Elston CW and Blamey RW (1986) Survival patterns in endocrine-treated advanced breast cancer. *Br J Surg* **73**: 752